# In Vitro Electrochemical Detection of Hydrogen Peroxide in Activated Macrophages via a Platinum Microelectrode Array

**DOI:** 10.3390/s21165607

**Published:** 2021-08-20

**Authors:** Victor M. Carriere, Jolin P. Rodrigues, Chao Tan, Prabhu Arumugam, Scott Poh

**Affiliations:** 1 Biomedical Engineering Department, Louisiana Tech University, Ruston, LA 71272, USA; carrierevictor7@gmail.com (V.M.C.); jpr041@latech.edu (J.P.R.); 2 College of Engineering and Science-Chemistry, Louisiana Tech University, Ruston, LA 71272, USA; 3 Institute for Micromanufacturing, Louisiana Tech University, Ruston, LA 71272, USA; waynetan019@gmail.com (C.T.); parumug@latech.edu (P.A.)

**Keywords:** reactive oxygen species (ROS), inflammation, RAW 264.7, macrophages, chronoamperometry, platinum microelectrode array, oxidative stress, electrochemical sensors

## Abstract

Oxidative stress, an excess of endogenous or exogenous reactive oxygen species (ROS) in the human body, is closely aligned with inflammatory responses. ROS such as hydrogen peroxide (H_2_O_2_), superoxide, and radical hydroxyl ions serve essential functions in fighting infection; however, chronic elevation of these species irreversibly damages cellular components. Given the central role of inflammation in a variety of diseases, including Alzheimer’s disease and rheumatoid arthritis, a low-cost, extracellular, non-invasive assay of H_2_O_2_ measurement is needed. This work reports the use of a platinum microelectrode array (Pt MEA)-based ceramic probe to detect time- and concentration-dependent variations in H_2_O_2_ production by activated RAW 264.7 macrophages. First, these cells were activated by lipopolysaccharide (LPS) to induce oxidative stress. Chronoamperometry was then employed to detect the quantity of H_2_O_2_ released by cells at various time intervals up to 48 h. The most stimulatory concentration of LPS was identified. Further experiments assessed the anti-inflammatory effect of dexamethasone (Dex), a commonly prescribed steroid medication. As expected, the probe detected significantly increased H_2_O_2_ production by LPS-doped macrophages, subsequently diminishing the pro-inflammatory effect in LPS-doped cells treated with Dex. These results strongly support the use of this probe as a non-invasive, robust, point-of-care test of inflammation, with a high potential for multiplexing in further studies.

## 1. Introduction

Reactive oxygen species (ROS)—principally superoxide (•O_2_^-^), radical hydroxyl ion (•OH), and nonradical hydrogen peroxide (H_2_O_2_)—are present in nearly all aerobic cell types [1]. When reactive radicals such as superoxide and nitric oxide hybridize, they form peroxynitrite (ONOO^-^), which is a highly reactive oxidizing agent, more toxic than the parent species. Peroxynitrite (ONOO^-^) and nitric oxide (•NO) are types of reactive nitrogen species (RNS), which, together with ROS, may transiently exceed the capacity of the body to remove them—a condition known as oxidative stress [2]. Oxidative stress is associated with numerous adverse health effects [1,2,3]. At the cellular level, these ill effects include irreversible oxidation of DNA, lipids, and amino-acid side chains (e.g., protein carbonylation) [1]. However, ROS/RNS species play a central role in essential physiological functions, such as the activation of macrophages to deploy ROS in fighting infection [4]. This ROS-mediated “respiratory burst” is a key component of the inflammatory response necessary for the regulation of cell growth and survival.

Despite the short-term benefit of this immune response, chronic inflammation is characteristic of diseases such as cancer, atherosclerosis, rheumatoid arthritis, Parkinson’s disease, and Alzheimer’s [1,2,4,5]. Chronic inflammation involves a state of prolonged oxidative stress in which the normal mechanisms of inflammatory resolution are impaired or do not function, such as the recruitment of anti-inflammatory M2 macrophages [6]. Chronically elevated levels of ROS, then, may have far-reaching detrimental effects in the body [7].

**Figure 1:**
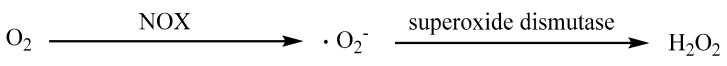
Conversion of free oxygen to superoxide radical species and hydrogen peroxide through the enzymes NOX and SOD.

Hydrogen peroxide is one of the primary redox signals in the body [8]. It is a non-radical product of the superoxide dismutase SOD-catalyzed dismutation of superoxide, which is produced continuously in mitochondria and elsewhere [9]. The most prevalent sources of endogenous superoxide are the electron transport chain of mitochondria, which generates superoxide radicals and occasionally hydrogen peroxide. Superoxide is generated and released by the NADPH oxidase (NOX) complexes as shown in Figure 1 [3]. Studies have shown that dual oxidase (DUOX) enzymes and the isoform NOX4 can produce H_2_O_2_ directly [10].

Much of the oxidative damage caused by hydrogen peroxide occurs indirectly, e.g., through the Fenton reaction in the presence of ferrous iron and the subsequent production of hydroxyl radicals [11]. Elevated H_2_O_2_ levels are a hallmark of inflammation and oxidative stress [2]. Since H_2_O_2_ is the most stable ROS in vivo, H_2_O_2_ is a suitable choice of biomarker in an assay of inflammation [12].

Current methods to detect and quantify oxidative stress, including ROS and RNS and their reactive intermediates, include spectroscopic methods, fluorescent-dependent methods [8,13], the use of chemiluminescent probes [14,15], spectrophotometric methods [16], chromatographic methods [17], and electrochemical sensors [18]. Each of these methods suffer from limitations such as the quenching of fluorescence when fluorescent probes or dyes are used and their inability to penetrate deeper layers of tissue. More recently, Vaneev et al. [19] reported in vitro and in vivo electrochemical measurement of ROS using an intracellularly placed platinized nanoelectrode. Although in vitro work appeared promising, the detection was somewhat invasive, having to pierce the cellular membrane, possibly compromising membrane integrity. This method also appeared to be impractical in the case of ROS detection in tissues, where the thickness of the tissue sample would provide inaccurate results.

The Amplex Red reagent is commonly used as an extracellular indicator of H_2_O_2_ production. Amplex Red is oxidized by horseradish peroxidase (HRP) in the presence of H_2_O_2_ to form resorufin, a fluorogenic molecule [20]. This assay is highly specific for H_2_O_2_ and, because H_2_O_2_: resorufin stoichiometry is 1:1, may be used to quantify extracellular H_2_O_2_ production [20]. However, similar to all fluorescent molecules, resorufin is subject to photobleaching with the subsequent attenuation of fluorescence. This tendency presents a practical problem in the design of an assay of inflammation.

The use of chronoamperometry in the detection of H_2_O_2_ and other ROS/RNS released by a small population of RAW 264.7 cells in a Pt MEA-containing microfluidic chamber has been demonstrated [21]. However, the intricacy of such a design may be of limited utility in a medical laboratory.

On the other hand, the ready availability of ceramic Pt MEA probes may permit the development of a more rapid scalable assay of extracellular H_2_O_2_. Furthermore, the ease of modifying the microelectrode surface allows for a wide range of multiplexed applications demonstrated from our group: Hossain et al. report the fabrication of a multiplexed glutamate/gamma-aminobutyric acid (GABA) Pt MEA probe via the addition of glutamate oxidase (GlOX) and GABase to separate microelectrodes within the same probe; this probe was used to detect ex vivo release of GABA in hippocampal rat brain slices [22]. Scoggin et al. report the use of GlOX-modified Pt MEA probes in vitro to detect glutamate uptake in astrocytes versus glioma cells [23].

In our study, we employ a similar approach to Vaneev [19] and Hossain and Scoggin [19,22,23], using a Pt MEA probe in an extracellular detection strategy. The immediate goal of this project is the non-invasive post hoc detection of stable concentrations of H_2_O_2_ released by a large population of macrophages and accurately detectable above baseline levels. Hence, real-time detection of H_2_O_2_ production by a small population of cells, as in Amatore et al. [21], was not attempted. Instead, H_2_O_2_ release was induced as follows: 1 × 10^5^ RAW 264.7 cells were incubated with varying concentrations of LPS for 6–48 h before testing for 4–8 min with the probe, to capture the electrochemical signals over different time periods and durations of probing. Sies et al. [8] reported the normal intracellular physiological H_2_O_2_ concentration as 1–10 nM in human liver, with extracellular concentrations being 100-fold. As our study was based on cell monolayer culture, our detection range planned to cover the oxidative eustress and distress ranges of the RAW 264.7 cells, with the expectation that the level of cellular complexity would be much lower in this study, enabling adequate detection.

To assess the feasibility of the Pt MEA as an H_2_O_2_ assay, the following hypotheses were investigated: (1) the probe will sense H_2_O_2_ over a physiologically relevant linear range (1–10 µM), (2) RAW 264.7 cells will produce H_2_O_2_ in response to LPS doping, (3) the probe will detect significantly more H_2_O_2_ in culture medium in LPS-doped samples than in untreated controls, (4) LPS-stimulated H_2_O_2_ production will be greater at the physiologically relevant temperature of 37 °C than at 4 °C, (5) dexamethasone, an anti-inflammatory steroid, will attenuate H_2_O_2_ production in LPS-doped samples, and (6) LPS doping and the operation of the probe will not adversely affect cell viability.

## 2. Materials and Methods

### 2.1. Fabrication and Preparation of Pt MEA Probes

**Figure 2:**
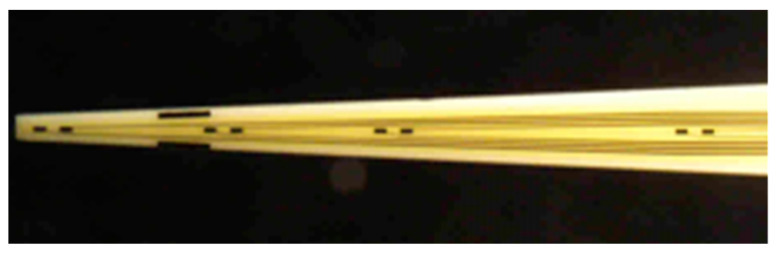
Detail of 8-TRK probe used in this project (CenMeT, University of Kentucky, Lexington, KY, USA). Each recording site is 50 × 100 µm. Recording site pairs have 100 µm separation. The spacing between recording site pairs is 1 mm, 1 mm, and 2 mm [25].

All Pt MEA probes were fabricated at the Center for Microelectrode Technology (CenMeT, University of Kentucky, Lexington, KY, USA) in conjunction with Thin Film Technology, Inc. (Buellton, CA, USA). Fabrication was performed as follows: a ceramic wafer (0.005 ± 0.0005 in. thick) was cleaned with sulfuric acid and chromium trioxide, rinsed with deionized water, and dried at 120 °C [24]. Photoresist was evenly spun over the whole surface of the ceramic wafer. A mask was used to etch the design of the microelectrode recording sites, bonding pads, and wires onto the photoresist [24]. Upon development of the photoresist, this design was exposed. An adhesion layer of titanium (500 Å), followed by the layer of platinum (1500 Å) that would comprise the Pt MEA, was coated onto the printed circuit [24]. Each ceramic wafer contained 56 microelectrodes. Upon diamond cutting of the wafer into discrete probes, each new probe contained eight platinized, platinum microelectrodes (100 µM × 50 µM) in four pairs shown in Figure 2 [25].

### 2.2. Cleaning

The probes were cleaned first in methanol and then electrochemically cleaned in acid. A size-exclusion layer of m-phenylenediamine (mPD) that rejects larger sized molecules was electrochemically coated over the Pt MEAs to mitigate the effect of interferents and further improve selectivity. Firstly, the Pt MEA probe was immersed in 0.05 M sulfuric acid in a two-electrode setup. In lieu of the Ag/AgCl reference electrode used in ROS assays, the more robust saturated calomel electrode (SCE) was used. A Gamry Reference 600+ potentiostat (Gamry Instruments, Warminster, PA, USA) was used to cycle one Pt microelectrode 15 times between −0.3 V and +1.0 V with a scan rate of 20 mV/s. Each of the remaining microelectrodes was cycled in this manner. The purpose of this acid cleaning is to increase H_2_O_2_ sensitivity via increasing the electrocatalytic sites by electrochemically etching the Pt grain boundaries and significantly reducing the charge transfer resistance of the Pt grains [25].

Secondly, the Pt MEA was immersed in a nitrogen-purged, 10 mM solution of mPD in 1 M NaCl. The same Pt MEA/SCE two-electrode setup described above was used, and each of the microelectrodes was continuously cycled between +0.2 V and +0.8 V, with a scan rate of 50 mV/s, for 40 min to achieve a coating thickness of ~37 ± 2.4 nm (mean ± SEM, *n* = 4). Upon completion of potential cycling, the probe was rinsed with deionized water and allowed to air-dry overnight.

### 2.3. Cell Culture and Probe Conditioning

RAW 264.7 cells (American Type Cell Collection, murine macrophage cell line) were maintained in Dulbecco’s Modified Eagle’s Medium (DMEM; VWR) with 4.5 g/L glucose, L-glutamine, and sodium pyruvate, supplemented with 10% FBS and 1% penicillin/streptomycin. Cells were cultured in 75 cm^2^ cell culture flasks (CellStar; T-75) and grown in a 37 °C, 5% CO_2_ incubator (VWR). Media was changed every 2–3 days or as needed: flasks were checked daily for notable color changes in the media. A pinkish-orange appearance indicated acidification by cellular waste products of the phenol red pH indicator present in DMEM. When the cells reached 80% confluency, they were seeded in 24-well plates at a density of 1 × 10^5^ cells/mL (for other plate types, seeding density was scaled up or down according to the surface area of the plate wells). 

### 2.4. Treatment of Cells with Lipopolysaccharide or Dexamethasone

After 24 h, the confluency of the cells was assessed under the inverted light microscope. When the cells were 50% confluent, they were doped with lipopolysaccharides (LPS) and/or dexamethasone (Dex) and placed in the incubator. RAW 264.7 cells were stimulated with varying concentrations of LPS (Enzo LifeSciences, Farmingdale, NY, USA) derived from Escherichia coli strain EH100 (Ra mutant). Cells were also treated with varying concentrations of Dex (Alfa Aesar, Haverhill, MA, USA). Both reagents were added via one of two methods:

#### 2.4.1. Method 1

Beginning with stock solutions of LPS or Dex and diluting separately in complete DMEM warmed to 37 °C, a 2X working solution of each desired final concentration was prepared. After aspirating spent media from each well, cells received, e.g., for LPS-only groups, 0.5 mL 2X LPS solution and 0.5 mL DMEM.

#### 2.4.2. Method 2

Working solutions (10X) of each desired final concentration of LPS or Dex were prepared in 1X phosphate buffered saline (PBS). 100 µL of media from each well was aspirated and replaced with an equivalent volume of 10X LPS, 10X Dex, or PBS. Wells receiving LPS and Dex had 200 µL media removed and replaced by 100 µL of each of the respective solutions.

### 2.5. Setup and Use of the Probe

#### 2.5.1. Calibration in the Presence of H_2_O_2_

**Figure 3:**
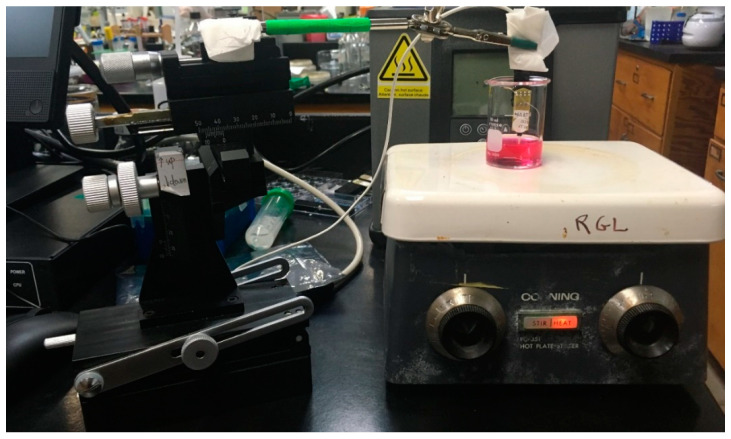
Experimental setup of the probe during calibration. The magnetic stirrer ensured a continuous steady flow of H_2_O_2_ to the electrode surface.

**Figure 4:**
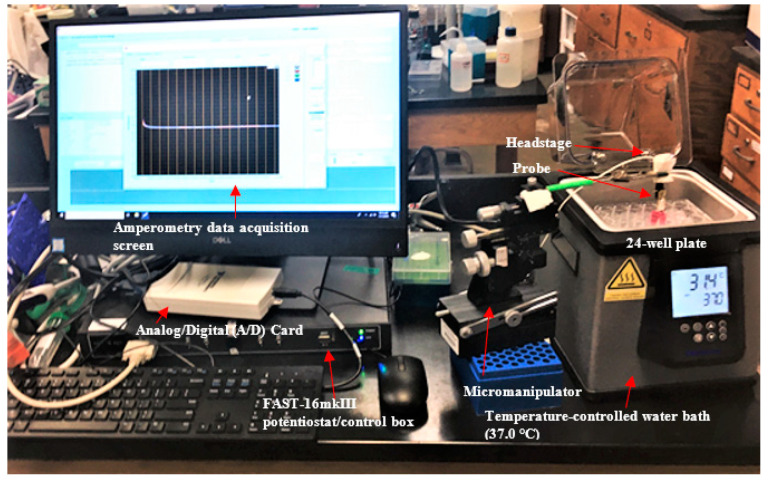
Setup of probe during amperometry experiments (+0.7 V vs. Ag/AgCl reference electrode). Data are recorded at a frequency of 10 Hz and displayed in real time as current (nA) vs. time (s).

As shown in Figure 3 and Figure 4, an 8-TRK probe was affixed to the head stage, which was connected to the Fast Analytical Sensing Technology (FAST) potentiostat/control box (FAST-16mkIII, Quanteon, Lexington, KT, USA) and submerged in 10 mL DMEM in a 20 mL beaker, as shown in Figure 3, to complete the calibration setup. A stirring hot plate (Corning, Corning, NY, USA) and micro magnetic stir bar were also used to ensure continuous steady transport of H_2_O_2_ to the Pt MEA surface. While the media was stirred at a constant rate, amperometry was performed as described below. When the slope of the current readout reached a sufficiently flat baseline (<|2| pA/min), increasing volumes of a 2 mM solution of stabilized H_2_O_2_ (Sigma, Darmstadt, Germany) were aliquoted to the beaker, and the corresponding increases in current were noted. Sensitivity (pA/µM) was derived from the slope of the calibration curve. 

Prior to each amperometry experiment, the probe was calibrated via the stepwise addition of H_2_O_2_ to cell culture medium. Abnormally large baseline currents obtained during calibration were assumed to reflect degradation of the mPD coating. Much empirical evidence established that the selectivity of such probes was compromised; 100 pA was selected as the baseline current above which probes should be discarded and replaced.

#### 2.5.2. Detection of Extracellular H_2_O_2_

Each 24-well plate was removed from the incubator and partially immersed in a 37 °C water bath (VWR). The Pt MEA was secured to a manual micromanipulator (World Precision Instruments), which was positioned such that the probe was inserted into the culture medium and secured 5 mm above the bottom surface of the plate as shown in Figure 4. Using the mPD-coated platinum microelectrode array (Pt MEA) with the FAST-16mkIII potentiostat in a two-electrode configuration, we performed chronoamperometry at an applied potential of +0.7 V with reference to an Ag/AgCl electrode. Current measurements were taken at a frequency of 10 Hz. The FAST system software (Quanteon) was used in all chronoamperometry experiments.

As proof of concept for detection of H_2_O_2_ release, RAW 264.7 cells were incubated at 37 °C, 5% CO_2_ until they reached ~50% confluency, then doped with 200 ng or 500 ng LPS. After further incubation for 24 h under the same conditions, the cells were probed. 

## 3. Results and Discussion

### 3.1. Calibration of the Probe in the Presence of H_2_O_2_

**Figure 5:**
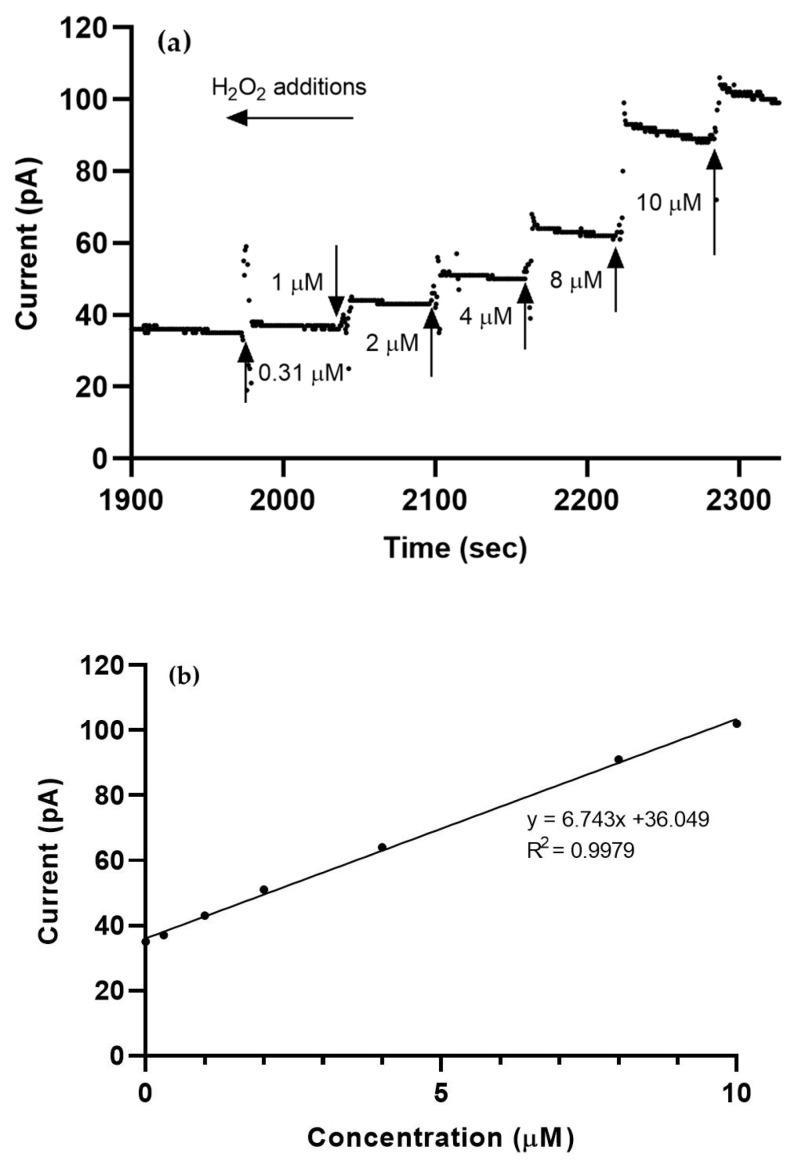
Representative graph of probe calibration in the presence of H_2_O_2_. (**a**) Increasing volumes of 2 mM H_2_O_2_ were added at sixty-second intervals such that the following final concentrations were reached: 0 µM, 0.31 µM, 1 µM, 2 µM, 4 µM, 8 µM, and 10 µM. (**b**). A linear fit of the current detected at each calibration step yielded a sensitivity of 6.743 pA/µM (R^2^ = 0.9979).

Calibration curves are shown in Figure 5; amperometry was performed in 10 mL of continuously stirred DMEM (+0.7 V voltage step versus Ag/AgCl; recording frequency: 10 Hz). H_2_O_2_ was aliquoted into the stirred media at 60 s intervals, as shown in Table 1, over the physiologically relevant H_2_O_2_ range of 1–10 µM. Probe calibration in this range ensured that H_2_O_2_ levels for monolayer cells before as well as after LPS-induced oxidative stress were capable of reliable detection by this probe, as supported by limited, but similar studies showing linearity of the standard curves at such low extracellular concentrations and periods of measurement [26,27]. 

In the example calibration shown in Figure 5, noise is seen at points of H_2_O_2_ addition (e.g., at 1980s in Figure 5a) and due to disturbances in the media (e.g., at 2030–2040s). However, this is a typical characteristic feature of the probe calibration curve and is expected. 

Care was taken to minimize background noise during testing. The analysis of experimental data was automated in MATLAB; briefly, random noise was filtered via the averaging of detected current at fifty evenly spaced time points across the last minute of testing. Additionally, the use of cell-free controls in each experiment helped ensure that noise of a longer duration would not artifactually increase the current and hamper accurate measurement.

### 3.2. Optimization of LPS and H_2_O_2_ Doping Protocol

To test the ability of the probe to detect H_2_O_2_ released by LPS-doped activated macrophages, 1 × 10^5^ cells/well were seeded in 24-well plates and incubated for 24 h. When the cells were 50% confluent, they were doped with 200 ng/mL or 500 ng/mL LPS according to Method 1 (see Method 1), incubated for 24 h, and tested. The following controls were included in this experiment: For negative controls, there were media with cells (media/cells) only (no doping of LPS). For positive controls, there were (a) media/cells with H_2_O_2_ (2 min), 2 µL of stock H_2_O_2_ was aliquoted two minutes before testing into wells containing media and cells (final concentration in well: 4 µM) and (b) media/cells with H_2_O_2_ (24 h), 2 µL of stock H_2_O_2_ was aliquoted 24 h before testing (i.e., at the time of LPS addition) to wells containing media and cells. This control was included to ensure that a known concentration of exogenous H_2_O_2_ would remain stable enough in solution to be detected by the probe. The difference in signal between both positive controls would be used to assess the stability of extracellular H_2_O_2_ released by LPS-doped cells. 

**Figure 6:**
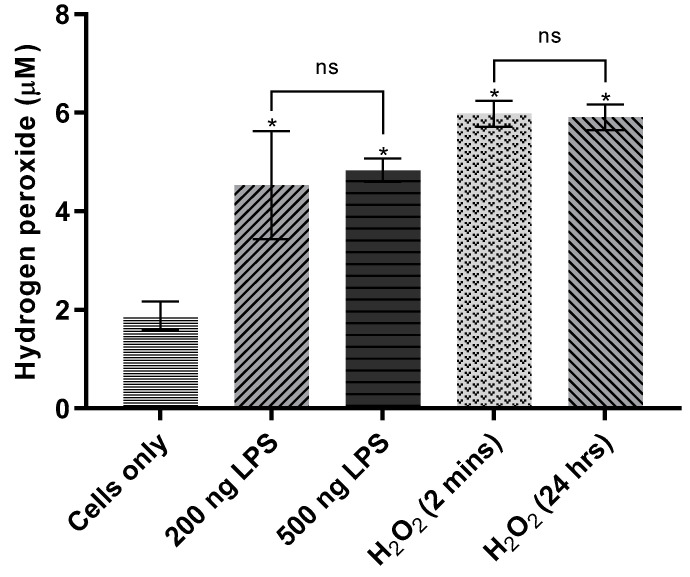
Effect of lipopolysaccharides on hydrogen peroxide production in RAW 264.7 cells. Cells were seeded in 24-well cell culture plates at a density of 1 × 10^5^ cells/well. Upon reaching ~50% confluency, treated cells were spiked with LPS in two concentrations (200 ng/mL and 500 ng/mL, in triplicate), then incubated for 24 h. After calibration of the Pt-MEA against H_2_O_2_ in complete cell culture medium (DMEM), amperometry was performed in each well for four minutes with a potential step of +0.7 V against an Ag/AgCl reference electrode. The sensitivity obtained during calibration (9.975 pA/µM; R^2^ = 0.9995) was used to translate current for each experimental condition to normalized H_2_O_2_ concentration (µM). Data shown correspond to Electrode 1 (E1), the electrode nearest the cells. One-way ANOVA (*n* = 5, *p* = 0.002) was performed to assess the significance of differences among all group mean concentrations, followed by the Tukey–Kramer test to assess pairwise significant differences (*n* = 5, * *p* < 0.05).

To assess the efficacy of this protocol, the following experiment was performed: RAW 264.7 cells were seeded in 24-well plates, as above, incubated for 24 h, and doped with 200 ng/mL or 500 ng/mL LPS. The resulting data showed an enhanced H_2_O_2_-generating response in LPS-doped cells, with significantly elevated H_2_O_2_ production in both 200 ng/mL (*p* = 0.039) and 500 ng/mL-treated (*p* = 0.021) cells (Figure 6). H_2_O_2_ produced was not significantly different between the two groups.

As shown in Figure 6, H_2_O_2_ showed apparent persistence in the 24-h positive control: currents generated (i.e., H_2_O_2_ detected) at E1 in the H_2_O_2_ (2 min) and H_2_O_2_ (24 h) groups were not significantly different (*p* = 0.99998). The normalized H_2_O_2_ concentration in both positive controls was ~6 µM—consistent with the expected ~4 µM increase above average H_2_O_2_ concentration in the cells-only negative control (1.88 ± 0.30 µM). Given the possibility of macrophages metabolizing external H_2_O_2_ to concentrations below the LOD of the probe [27], the similar signals obtained in both H_2_O_2_-positive controls indicate a robust ability to detect H_2_O_2_ incubated for long periods with live cells. This result extended to LPS-doped cells as well. Taken together, these two results validate the hypothesis that stable extracellular concentrations of LPS-induced H_2_O_2_ may be detected in RAW cells via the probe. 

### 3.3. LPS Dosage Concentration for H_2_O_2_ Detection 

**Figure 7:**
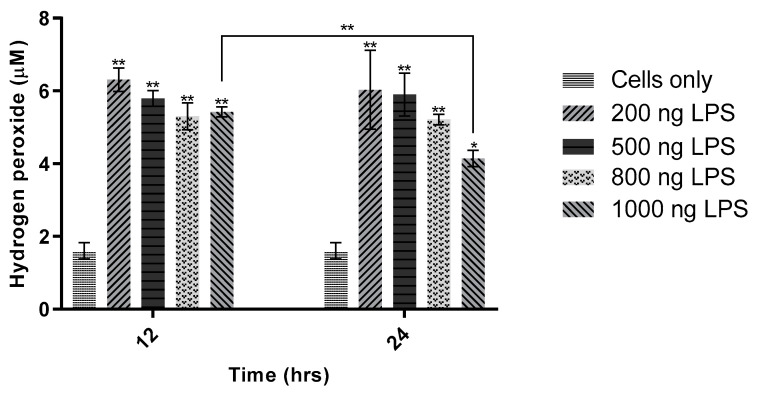
Effect of lipopolysaccharides concentration on hydrogen peroxide production in RAW 264.7 cells. Four concentrations of H_2_O_2_ (200 ng/mL, 500 ng/mL, 800 ng/mL, and 1000 ng/mL) were added to cells in triplicate. Cells were incubated for 12 or 24 h. The sensitivity obtained was 4.10 pA/µM (R^2^ = 0.9995). Data shown to correspond to Electrode 1 (E1), the microelectrode nearest the cells. One-way ANOVA (*n* = 14, *p* < 0.001) and the Tukey–Kramer test (*n* = 14, * *p* < 0.05, ** *p* < 0.001) were used to assess statistical significance.

**Figure 8:**
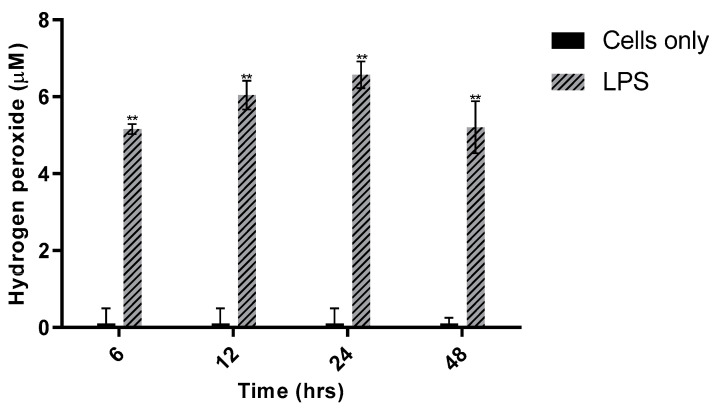
Effect of incubation time on H_2_O_2_ production in RAW 264.7 cells. An amount of 200 ng/mL LPS was added to cells plated in triplicate in 24-well plates (seeding density: 1 × 10^5^ cells/mL). Cells were incubated for 6, 12, 24, or 48 h. Probe sensitivity: 11.0 pA/µM (R^2^ = 0.9997). One-way ANOVA (*n* = 21, *p* < 0.001) and the Tukey–Kramer test (*n* = 21, ** *p* < 0.001) were used to assess statistical significance.

Figure 7 is to show the optimal LPS concentration for extracellular H_2_O_2_ production; therefore, only two selected concentrations of LPS were included in the next experiment. RAW 264.7 cells were seeded in 24-well plates as above and doped with LPS in the following (final) concentrations: 200 ng/mL, 500 ng/mL, 800 ng/mL, and 1000 ng/mL. The cells were incubated for 12 or 24 h before testing. H_2_O_2_ production in RAW 264.7 cells showed no significant LPS dose dependence (Figure 8), implying that the range of LPS concentrations assayed, 200 ng/mL^–1^ µg/mL, is saturating yet sublethal. Extracellular H_2_O_2_ production was more pronounced at the two lower concentrations of LPS (Figure 7). The apparent stability of extracellular H_2_O_2_ was further supported by this experiment: between 12 h and 24 h, only the highest dosage of LPS (1 µg/mL) showed any statistically significant reduction in H_2_O_2_ detected by the probe, whereas the lower concentrations remained relatively stable over this period. Given the toxicity of LPS and the potential concomitant reduction in cell viability [28], the lowest dose, 200 ng/mL, was selected as the best concentration for further study.

### 3.4. Incubation Time Study

Figure 8 summarizes time studies that were performed to discern the effect of incubation time on H_2_O_2_ production. The optimal LPS concentration of 200 ng/mL was used. RAW 264.7 cells were seeded in 24-well plates as described previously, then doped with 200 ng/mL LPS and incubated for 6, 12, 24, or 48 h.

At each of the four time points, H_2_O_2_ was significantly elevated in LPS-treated cells versus cell-only controls (*p* < 0.001). Somewhat unexpectedly, the LPS-stimulated H_2_O_2_ detected was not statistically different across the four time points. This prolonged stability indicated that 200 ng/mL LPS produced a stable, saturating concentration of extracellular H_2_O_2_. While comparing these incubation timepoints, LPS stimulation of the cells at all these timepoints exhibits statistically similar [H_2_O_2_] release. Thus, selecting an optimal LPS incubation time was somewhat arbitrary; therefore, one or more of these incubation timepoints were chosen for subsequent experiments.

### 3.5. Incubation Temperature Study

The inflammatory response depends on metabolically functioning cells, which must initiate a host of synthetic and transport processes. In order to determine that the H_2_O_2_ measured is due to cellular processes, LPS-doped cells were incubated at 4 °C. The functioning of mammalian cells in vitro depends strongly on incubation temperature, it was expected that H_2_O_2_ production in LPS-doped RAW cells incubated at 4 °C would be near zero. 

**Figure 9:**
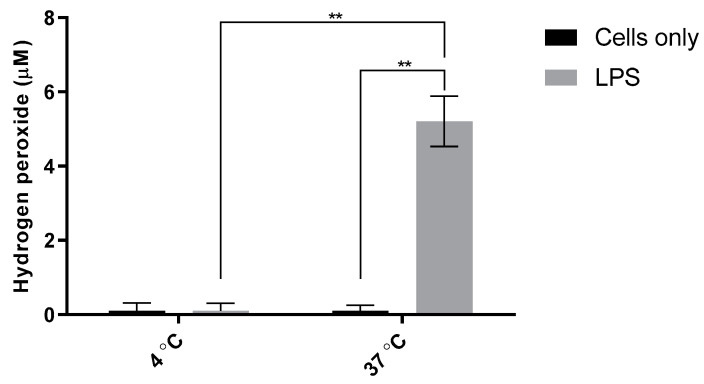
Effect of reduced incubation temperature on H_2_O_2_ production in RAW 264.7 cells. LPS-doped cells received 200 ng/mL in each well. Cells were incubated for 48 h at 4 °C or 37 °C (5% CO2) following addition of LPS. Probe sensitivity: 11.0 pA/µM; R^2^, 0.9997. One-way ANOVA (*n* = 8, *p* < 0.001) and the Tukey–Kramer test (*n* = 14, ** *p* < 0.001) were used to assess statistical significance.

To assess the effect of reduced incubation temperature on H_2_O_2_ production, RAW 264.7 cells were seeded in 24-well plates as described above. All plates were initially incubated at 37 °C, 5% CO_2_ to ensure the cells could adhere and proliferate. After doping the cells with 200 ng/mL LPS, cells were incubated at either 4 °C or 37 °C for 48 h. Subsequent testing with the ROS probe revealed the expected lack of extracellular H_2_O_2_ produced by 4 °C-incubated cells as shown in Figure 9.

H_2_O_2_ was significantly elevated when LPS-doped cells were incubated at 37 °C, whereas little or no H_2_O_2_ were detected for cells incubated at 4 °C, thus supporting the metabolic energy dependence of H_2_O_2_ production in LPS-activated macrophages. 

### 3.6. Effect of Dexamethasone on H_2_O_2_ Production

**Figure 10:**
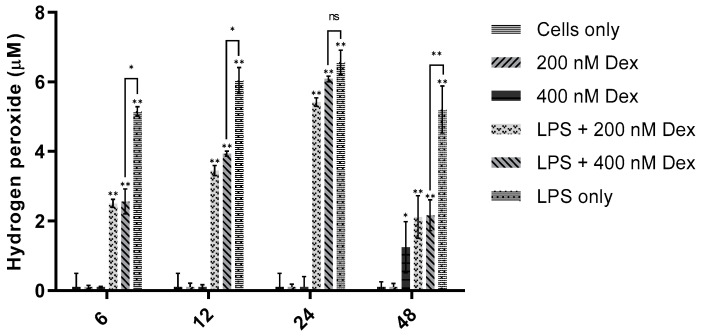
Effect of dexamethasone on H_2_O_2_ production in RAW 264.7 cells. Cells received 200 ng/mL LPS and/or 200 nM or 400 nM Dex in each well. Cells were incubated for 6, 12, 24, or 48 h following addition of LPS and/or Dex. Probe sensitivity: 11.0 pA/µM; R^2^, 0.9997. One-way ANOVA (*n* = 14, *p* < 0.001) and the Tukey–Kramer test (*n* = 14, * *p* < 0.05, ** *p* < 0.001) were used to assess statistical significance.

Figure 10 shows the effect of an anti-inflammatory drug on H_2_O_2_ production. It was expected that the anti-inflammatory glucocorticoid dexamethasone (Dex) would counter the action of LPS, thus reducing cumulative H_2_O_2_ production in LPS-doped RAW 264.7 cells [29]. To assess the effect of treating cells with Dex in conjunction with LPS, cells were treated with 200 ng/mL LPS and either 200 nM or 400 nM Dex, then incubated for 6, 12, 24, or 48 h.

At each of the four time points, cells treated with LPS and Dex (200 nM and 400 nM) produced significantly more H_2_O_2_ than cell-only controls (*p* < 0.001). The H_2_O_2_ production effect was mitigated by Dex at three time points: at 6, 12, and 24 h after treatment, LPS + Dex groups produced significantly less H_2_O_2_ than the LPS-only positive control (6 h and 12 h: *p* < 0.05; 24 h: *p* < 0.001). Between 6 and 24 h, these data appear to show the anti-inflammatory action of Dex being gradually outpaced by LPS-stimulated H_2_O_2_ production. Yet, 48 h after treatment, the anti-inflammatory, ROS-suppressive effect was strongest (*p* < 0.001). Unexpectedly, Dex alone stimulated significant H_2_O_2_ production (*p* < 0.05) at 48 h. This seeming paradox should be investigated further. Despite the imperfect trend in the data, these results support that the probe may be used as an extracellular assay not just of H_2_O_2_, but for inflammation in general.

### 3.7. Viability Assay of LPS-Doped and ROS-Probed Cells

In order to assess the viability of cells after LPS stimulation, an MTT assay kit (Promokine) was used. Cells were cultured in 96-well plates and subjected to one of the following conditions: (1) negative control, (2) 200 ng/mL LPS, (3) 500 ng/mL LPS, (4) exposure to the ROS probe (+0.7 V against Ag/AgCl) for 5 min, (5) exposure to the ROS probe (+0.3 V against Ag/AgCl) for 5 min. 

**Figure 11:**
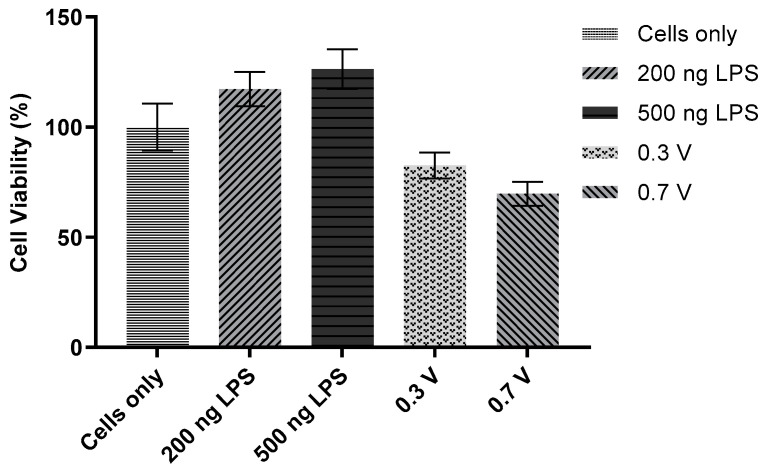
Effect of LPS or ROS probe operation on viability of RAW 264.7 cells. 96-well plates were seeded with 1 × 10^4^ RAW 264.7 cells, grown to 50% confluency, and doped with 200 ng/mL or 500 ng/mL LPS, or probed at one of two potential steps: +0.7 V and +0.3 V. One-way ANOVA (*n* = 6, *p* = 0.0029) and Tukey–Kramer post hoc analysis (*n* = 6) were performed.

With both 200 ng/mL and 500 ng/mL LPS, RAW 264.7 cells exhibited viability slightly greater than 100% (*p* > 0.05): LPS appears to encourage proliferation at these doses as shown in Figure 11. This result agrees with a phenomenon observed in the inverted microscope: after incubation with LPS in 24-well plates, these cells often appeared more confluent than those of the negative control (data not shown).

Tukey–Kramer analysis of these results indicated that neither LPS doping nor use of the probe significantly reduced cell viability (*p* > 0.05). At both 200 ng/mL and 500 ng/mL, RAW 264.7 cells exhibited viability slightly greater than 100% (*p* > 0.05), LPS appears to enhance cell proliferation at these doses, supporting evidence in previous studies [9,30] and demonstrating that LPS can stimulate immune cell proliferation. This result agrees with a phenomenon observed in the inverted microscope: after incubation with LPS in 24-well plates, these cells often appeared more confluent than those of the cell-only control. This effect might be attributed to the greater number of cells accounting for some of the increase in cumulative H_2_O_2_ seen in LPS-doped groups. 

The reduction in viability at both voltage steps was just shy of statistical significance. However, in this assay, the probe was used in 100 µL, rather than 1 mL, of media (i.e., a 96-well plate, rather than a 24-well plate). It is possible that the closer confines of the 96-well plate caused additional stress to the cells when the probe was introduced and operated.

## 4. Conclusions

This work provides a robust demonstration of a non-invasive use of Pt MEA probes in an assay of inflammation. Stable concentrations of extracellular H_2_O_2_ released in vitro by activated macrophages placed under oxidative stress were detected up to 48 h after doping with LPS. The Pt MEA was used to quickly and quantitatively rapid measure the anti-inflammatory effect of Dex on LPS-doped RAW 264.7 cells. This experiment served as an in vitro model of an assay for inflammation.

Future work with this probe will involve the multiplexing of the probe to allow for additional ROS/RNS sensing (e.g., the simultaneous detection of H_2_O_2_ and nitric oxide with separate microelectrodes). Further modifications of the Pt MEA will allow such multiplexed sensing in conjunction with the monitoring of treatment (e.g., the depletion of therapeutic antioxidants such as ascorbic acid). This additional work would point toward the creation of a rapid, convenient, non-invasive, comprehensive clinical assay of oxidative stress and inflammation. 

## Figures and Tables

**Table 1:** Volume 2 mM H_2_O_2_ added during each calibration step, with a resultant final concentration of H_2_O_2_ in 10 mL media.

Step	H_2_O_2_ Added (µL)	[H_2_O_2_] (µM)
1	1.55	0.31
2	3.45	1
3	5	2
4	10	4
5	20	8
6	40	10

## Data Availability

Not applicable.
